# Efficacy and cost-effectiveness analysis of flexible ureteroscopic lithotripsy with TFDS in the treatment of urolithiasis

**DOI:** 10.3389/fsurg.2024.1489397

**Published:** 2024-11-27

**Authors:** Xiang Gao, Peng Han, Yiping Zong, Zijie Wang, Wei Zhang, Pei Lu

**Affiliations:** ^1^ Department of Urology, The Second Affiliated Hospital of Nanjing Medical University, Nanjing, China; ^2^ Department of Urology, The First Affiliated Hospital of Nanjing Medical University, Nanjing, China; ^3^ Department of Urology, The Affiliated Yixing Hospital of Jiangsu University, Yixing, China

**Keywords:** total flavonoids of desmodium styracifolium, FURL, cost-effective analysis, urolithiasis, efficacy & safety

## Abstract

**Background:**

At present, there is no effective drug to remove residual stones. Total flavonoids of desmodium styracifolium (TFDS) is an innovative traditional Chinese medicine listed in 2022, which can be used to treat ureteral calculi. This study was to explore the effectiveness and economic value of TFDS in the treatment of residual stones after flexible ureteroscopic lithotripsy (FURL).

**Methods:**

A total of 161 patients who underwent unilateral ureteroscopic lithotripsy for urinary calculi by the same surgeon in our center from May 2022 to February 2024 were retrospectively included. According to the use of stone-removal drugs after operation, patients were divided into TFDS group and Control group. The residual stones showed by x-ray plain film when the double J tube was removed were compared between the two groups, and the economic benefits of TFDS were analyzed by cost-benefit analysis.

**Results:**

The data of 161 patients were collected, including 80 cases in TFDS group and 81 cases in Control group. The SFR rates at the endpoint of follow-up in TFDS group and Control group were 98.75% and 88.88%, respectively. In the subgroup analysis of post-operative residual stones, the stone clearance rate of TFDS was higher (47.62% vs. 18.18%). No obvious adverse events were reported in two groups. The cost/benefit ratio of TFDS was lower (20.43 vs. 32.57). Cost of TFDS was increased by ¥12.97 for each additional unit of total effective rate.

**Conclusion:**

The combination of dusting FURL and TFDS can effectively remove the urolithiasis when compared to only FURL, which showed highly economic benefits.

## 1 Introduction

Ureteroscopic lithotripsy is an important method for the treatment of urinary calculi with the advantages of less damage, less pain, high safety, quick recovery and wide indications (1). However, postoperative residual stones are one of the important reasons that plague surgeons and patients. Postoperative residual stones refer to the residual stones found on the surgical side after operation. Postoperative residual stones may lead to conflicts between doctors and patients, resulting in additional economic burden due to continued treatment. Therefore, effective treatment of postoperative residual stones and reducing the cost burden after the occurrence of residual stones are important solutions.

At present, the methods of treating postoperative residual stones include drug therapy and reoperation (2). In general, reoperation is considered only when the drug treatment is ineffective. However, there is no reliable high-level evidence of effective drug therapy. Total flavonoids of Desmodium styracifolium (TFDS) is a new innovative Chinese medicine listed in China, which can be used to treat ureteral calculi (3). It is reported that its active ingredients Lysimachia christinae Hance and flavonoids can dilate the ureter and promote the discharge of stones (3, 4). Furthermore, TFDS was observed to attenuate the formation of hydroxy-L-proline-induced calcium oxalate urolithiasis in rats, mainly through the prevention of oxidative stress changes and relieved apoptosis and autophagy of HK-2 cells damage induced by calculi (5, 6). Recently, drugs including TFDS have been applied in the clinical practice, which provide a novel approach for the expelling of ureter stones. However, its effect on the treatment of residual stones after ureteroscopic lithotripsy is still unknown.

In this study, a retrospective cohort study was designed to explore the efficacy of dusting flexible ureteroscopic lithotripsy (FURL) combined with TFDS in the treatment of urolithiasis. In addition, cost-effectiveness analysis was also performed to investigate the economic burden of this novel surgical protocol.

## 2 Patients and methods

### 2.1 Ethic statement and study population

All research programs and content involving human participants strictly comply with the provisions of the “World Medical Association Declaration of Helsinki” and “Istanbul Declaration”. With the approval of the Ethics Committee of the First Affiliated Hospital of Nanjing Medical University (2022-SRFA-429), the patient's informed consent was waived.

The retrospective cohort study was conducted on patients who underwent ureteroscopic lithotripsy in the same hospital from June 2022 to February 2024. The inclusion criteria include: (i) surgery performed by the same surgeon (Pei Lu); (ii) unilateral ureteroscopic lithotripsy; (iii) all stones were examined by composition analysis; (iv) patients over 18 years old; (v) Abdominal plain film examination on the second day after operation; (vi) At least one outpatient follow-up after discharge. Exclusion criteria include: (i) uric acid stones; (ii) lack of preoperative abdominal CT; (iii) additional surgical procedures at the same time; (iv) missing data. Appropriate consent and consent of participants and legal guardians/parents were obtained.

All patients were confirmed by preoperative CT to meet the indications of ureteroscopic lithotripsy and underwent standard operation. The next day after operation, abdominal plain film examination was performed. Abdominal plain film examination was performed again during postoperative outpatient follow-up to confirm the effect of drug stone removal and remove the ureteral stent.

### 2.2 Surgical procedures and follow-up

The operation steps of ureteroscopy are as follows: (i) the ureteroscope was used to enter the bladder. (ii) the loach guide wire entered the affected ureter. (iii) the ureteroscope entered the affected ureter along the loach guide wire. (iv) guide wire was retained, (v) the 10F/12 F flexible ureteroscope sheath was placed along the guide wire. (vi) the 8 F/9.8 F flexible ureteroscope enters the affected ureter to find the stone. (vii) a 365 µm holmium laser fiber with the settings of 30–44 W (0.8–1.5 J with 20–40 HZ) was used to disintegrate the calculi into dusts less than 2 mm. (viii) Finally, a ureteral stent was retained. For flexible ureteroscopic surgery, we used a 365 µm holmium laser fiber with the settings of 20–40 W (0.8–2.0 J with 10–20 HZ) to separate the calculi into fragments less than 5 mm and a stone basket was used during the operation to clean up the gravel, while no stone-removal treatment was administrated after the operation. In TFDS group, we carried out powdered lithotripsy during the operation, without the use of stone basket, and administrated drugs containing TFDS (GUANGSHITONG®, Wuhan Guanggu Renfu Biomedical Co., Ltd., Wuhan, China) 0.6 g tid orally to expel the stones after the operation, while patients in Control group did not take any lithagogue drugs after FURL procedures. Patients in both groups were required to visit outpatient clinic in our center at three to four weeks after discharge, and least follow-up time of patients was three weeks.

### 2.3 Data collection

The demographic characteristics of the patients were recorded, including age, gender, BMI, length of medical history. Preoperative CT and test indicators were reviewed, including bacterial culture, urinary protein, urinary leukocyte esterase, urinary leukocyte count, bacteria, alanine aminotransferase (ALT), aspartate aminotransferase (AST), urea, serum creatinine, white blood cells, lymphocyte count, neutrophil count, hemoglobin, stone location, stone maximum diameter, and CT value. The surgical records were reviewed, and the duration of operation, surgical side, and ureteral stenosis were extracted. The postoperative hospital stay, postoperative stone composition, postoperative medication, and stone-free rate (SFR) at the 1st post-operative day and out-patient follow-up were recorded. In order to follow up the outcome, we extracted the medical records of the patient postoperative outpatient visit, including the abdominal plain film at the time of follow-up, and reported adverse events. In addition, we also recorded the patient expenses, including hospitalization expenses and details and postoperative medication costs.

In this study, the SFR was calculated by the proportion of patients with residual stones less than 4 mm, which was reported by CT and/or KUB examination.

### 2.4 Statistical analysis

Efficacy was defined as no stone on x-ray during outpatient follow-up. R vision was used to analyze the data. The measurement data were expressed as Mean (SD) or Median (1st Quartile, 3st Quartile) according to normality. Nonparametric test or independent sample t test was used for comparison between groups. The enumeration data were expressed as a percentage. Chi-square test or Fisher exact probability test was used for comparison between groups. *P* value < 0.05 indicated that the difference was statistically significant.

The cost-effectiveness ratio (C/E) was used to evaluate the economic value of FURL combined with TFDS in the treatment of urolithiasis. Direct costs (such as health care expenses) and indirect costs (such as lost patient time) were identified and quantified, and Markov chain model was used to simulate transitions between different health states to reflect baseline conditions (7). C/E = (drug cost/total clinical effective rate), and the incremental cost-effectiveness ratio (△C/△E) between the higher cost scheme and the lower cost scheme was calculated to assess the cost-effectiveness of different options.

## 3 Results

### 3.1 Study population

**Figure 1:**
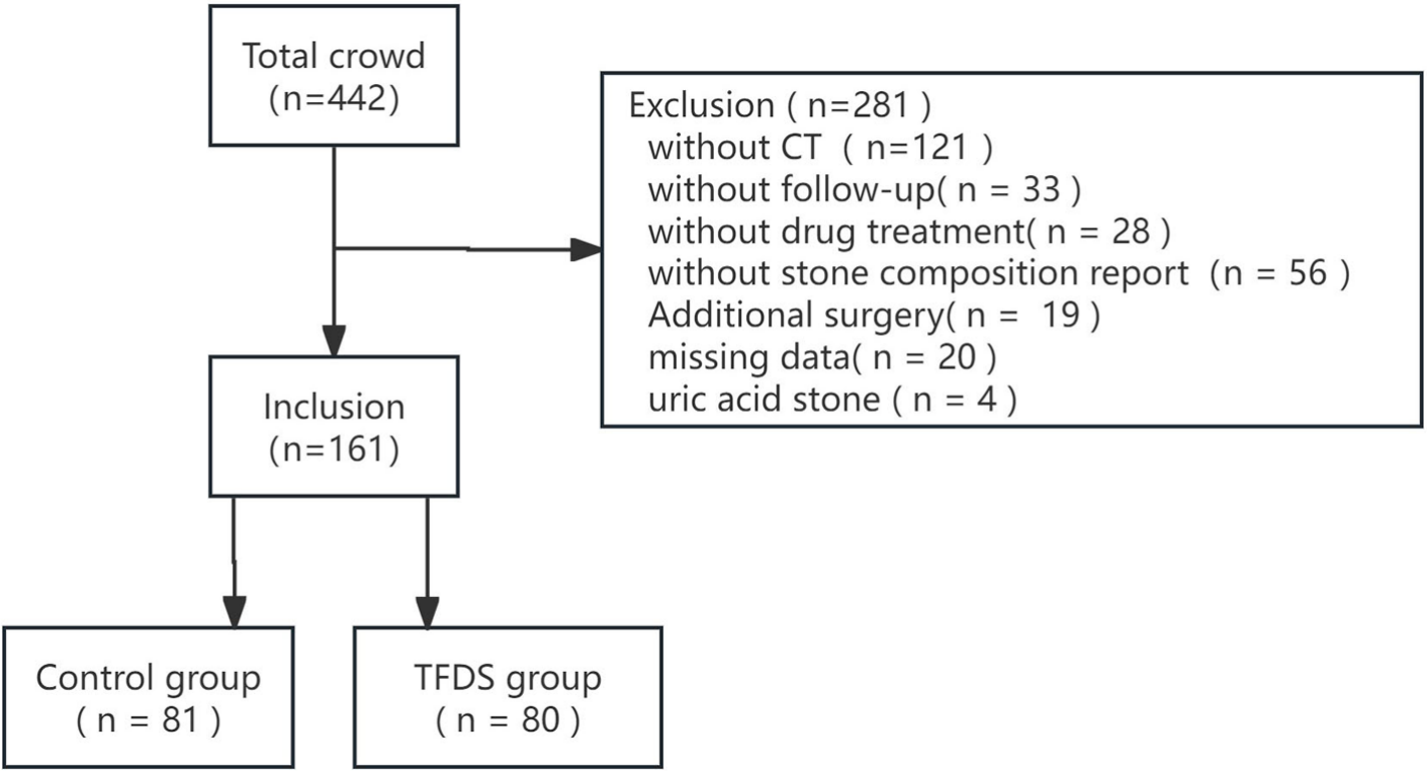
Flow diagram of patient enrollment.

The enrollment of patients is shown in Figure 1. A total of 161 patients were included, 81 in the Control group and 80 in TFDS group, with an average follow-up of 29 days. Table 1 shows the baseline characteristics of the study population. There was no significant difference in variables between the two groups.

**Table 1:** Comparison of demographic characteristics between the two groups.

Variables	Control group (*n* = 81)	TFDS group (*n* = 80)	*P* value
Age, years, mean (SD)	51.42 (12.93)	51.64 (11.09)	0.91
Gender, *n* (%)			0.90
Female	24 (29.63)	23 (28.75)	
Male	57 (70.37)	57 (71.25)	
BMI, *M* (*Q*₁, *Q*₃)	24.62 (22.49, 26.56)	24.94 (23.34, 26.60)	0.53
Urinary leukocyte,/μl, *M* (*Q*₁, *Q*₃)	35.00 (16.00, 104.00)	55.00 (23.75, 106.50)	0.15
ALT, U/L, *M* (*Q*₁, *Q*₃)	17.90 (13.50, 30.30)	21.30 (16.78, 29.20)	0.061
AST, U/L, *M* (*Q*₁, *Q*₃)	19.70 (17.70, 25.00)	22.15 (18.53, 28.22)	0.13
Urea, mmol/L, *M* (*Q*₁, *Q*₃)	5.43 (4.44, 6.67)	5.75 (4.58, 7.17)	0.17
Serum creatinine, umol/L, *M* (*Q*₁, *Q*₃)	75.80 (64.20, 89.40)	73.95 (61.48, 90.60)	0.78
Duration of stone disease history, days, *n* (%)			0.93
≤14	19 (23.46)	20 (25.00)	
15–30	16 (19.75)	14 (17.50)	
≥31	46 (56.79)	46 (57.50)	
Side, *n* (%)			0.57
Left	44 (54.32)	47 (58.75)	
Right	37 (45.68)	33 (41.25)	
Location of stones on CT, *n* (%)			0.82
Kidney	22 (27.16)	23 (28.75)	
Ureter	31 (38.27)	33 (41.25)	
Kidney and ureter	28 (34.57)	24 (30.00)	
Positive results of preoperative urine culture, *n* (%)	8 (9.88)	8 (10.00)	0.98
Leukocyte esterase in urine, *n* (%)			0.59
Negative	58 (71.60)	50 (62.50)	
1+	9 (11.11)	14 (17.50)	
2+	7 (8.64)	7 (8.75)	
3+	7 (8.64)	9 (11.25)	
Urine bacterial smear, *n* (%)			0.58
Negative	66 (81.48)	67 (83.75)	
1+	7 (8.64)	8 (10.00)	
2+	2 (2.47)	3 (3.75)	
3+	1 (1.23)	1 (1.25)	
4+	5 (6.17)	1 (1.25)	

Abbreviations: *M*, median; *Q*₁, 1st quartile; Q₃, 3st quartile; SD, standard deviation; BMI, body mass index.

### 3.2 Clinical efficacy and safety of dusting FURL and TFDS

Table 2 presents the comparison of operation-related information between the TFDS group and the Control group. To be noted, SFR on the first day after surgery in patients of TFDS group was significantly lower, of which increased remarkably at the endpoints of follow-up (TFDS vs. Control: First day after surgery, 57.50% vs. 72.84%, *P* = 0.030; Endpoints, 98.75% vs. 88.88%, *P* = 0.0095; Table 2). Moreover, total cost during hospitalization in TFDS group was statistically decreased (TFDS vs. Control: ￥22,981.30 vs. ￥26,619.2, *P* < 0.001; Table 2). In addition, no significant difference was observed in other variables.

**Table 2:** Results of operation-related information in TFDS and control groups.

Variables [*M*, (*Q*₁, *Q*₃)]	Control group (*n* = 81)	TFDS group (*n* = 80)	*P* value
SFR at 1st day after surgery, *n* (%)	59 (72.84)	46 (57.50)	**0**.**030**
SFR at follow-up, *n* (%)	72 (88.88)	79 (98.75)	**0**.**0095**
hospitalization length (day)	4.00 (3.00, 5.00)	3.50 (3.00, 5.00)	0.078
Operation duration (minute)	33.00 (21.00, 45.00)	35.50 (26.75, 45.50)	0.36
Stone long diameter (mm)	8.30 (6.20, 12.50)	8.30 (6.47, 10.05)	0.50
Stone short diameter (mm)	6.10 (4.30, 7.70)	6.15 (4.38, 7.32)	0.90
Stone longitudinal diameter (mm)	9.00 (6.00, 12.50)	9.10 (6.30, 13.10)	0.68
CT value (Hu)	983.00 (781.00, 1,243.00)	1,172.50 (879.75, 1,299.50)	0.075
Stone composition, *n* (%)			0.16
Calcium oxalate stone	63 (77.78)	69 (86.25)	
Apatite stone	18 (22.22)	11 (13.75)	
History of ureteroscopy on operative side, *n* (%)	8 (9.88)	5 (6.25)	0.40
Ureteral stenosis on operative side, *n* (%)	9 (11.11)	13 (16.25)	0.343
Placement of ureteral stent before operation, *n* (%)	8 (9.88)	13 (16.25)	0.23

Abbreviations: TFDS, total flavonoids of desmodium styracifolium; *M*, median; *Q*₁, 1st quartile; *Q*₃, 3st quartile; SD, standard deviation; BMI, body mass index; ESWL, extracorporeal shock wave lithotripsy.

The bold values represent the statistical significance in the analysis.

In order to extensively explore the effect of TFDS on stone removal, study population with postoperative residual stone (*n* = 43) was extracted for subgroup analysis (Supplementary Table S1), whom were further divided into patients administrated with TFDS (residual-TFDS group) and without TFDS (residual-Control group). No significant difference in baseline data, surgical information, and follow-up days were observed between two groups. Importantly, the residual stone removal rate of residual-TFDS group was significantly higher than residual-Control group (*P* = 0.039; Supplementary Table S1).

Since the previous literature mentioned the good therapeutic effect of TFDS on calcium oxalate stones, the calcium oxalate residual stone cohort was extracted for further subgroup analysis. Supplementary Table S2 showed that the TFDS group had a higher SFR (*P* = 0.003). Similarly, at follow-up, group without residual stones showed higher TFDS administration proportion (*P* = 0.003; Supplementary Table S3), lower preoperative AST (*P* = 0.037; Supplementary Table S3), and more severe preoperative urine bacterial detection (*P* = 0.042; Supplementary Table S3). Multivariate logistic regression showed that high TFDS use rate was a protective factor for successful removal of calcium oxalate residual stones (Supplementary Table S4).

Furthermore, no severe adverse events were reported during outpatient follow-up.

### 3.3 Cost-effectiveness analysis

As mentioned above, the total cost during hospitalization in TFDS group was significantly lower than the Control group, while the drug costs showed the opposite result. Considering the high SFR results of TFDS, a cost-effectiveness analysis was performed to evaluate the economic value of TFDS. C/E refers to the ratio of cost to effectiveness. The lower the C/E value is, the higher the economic value of the corresponding drug is. The results showed that the C/E value of the TFDS group was lower than that of the control group (17.99 vs. 28.59), while compared with the control group, the cost of TFDS increased by only ￥11.45 for each additional unit of total efficiency (Table 3). Sensitivity analysis is often used to measure the economic value of corresponding interventions when costs or benefits change. Assuming that the benefit is constant and the cost is reduced by 20%, the C/E value of TFDS is still lower than that of the control group (Supplementary Table S5).

**Table 3:** Results of cost-effective analysis in this study.

	Number	Median cost (C)/RMB	Effectiveness (E)/%	C/E	△C	△E	△C/△E
Control group	22	519.75	18.18	28.59			
TFDS group	21	856.80	47.62	17.99	337.05	29.44	11.45

Abbreviations: TFDS, total flavonoids of desmodium styracifolium; RMB, Renminbi.

## 4 Discussion

Urinary calculi are a common disease that plagues the global population and ranks first in urinary diseases. Its treatment mainly relies on active monitoring, medical expulsive therapy (MET), ultrasonic lithotripsy and surgical treatment. For patients undergoing surgical treatment, adjuvant oral medication is a common regimen, which can excrete residual stones in the ureter, reduce the risk of secondary surgery, and improve patient satisfaction. However, there is currently no ideal anti-lithiasis drug.

Common lithagogues include *α*-blockers, calcium channel antagonists, non-steroidal anti-inflammatory drugs, phosphodiesterase-5 inhibitors, β3 adrenergic receptor agonists, and traditional Chinese medicines. The latest RCT study showed that the use of tamsulosin for symptomatic urinary calculi less than 9 mm did not significantly increase the stone clean rate, although the relevant guidelines were recommended (8). Many studies have shown that the stone expulsion rate of tamsulosin is higher than that of nifedipine (9). Previous studies have found that diclofenac sodium can significantly alleviate renal colic, but compared with the control group did not increase the rate of stone discharge. Furthermore, it is indicated that comprehensive treatment including traditional Chinese medicine and tamsulosin, showed significantly higher efficacy and safety than the solely administration of tamsulosin for removing renal stones (10). Especially for the traditional Chinese medicine, it has been widely recognized for its less side effects, natural and synthetic conditioning efficacy, and individuation in the treatment of urolithiasis (11, 12). Therefore, the clinical study on the comparison and efficacy of traditional Chinese medicine is emerging.

China is one of the main areas of urolithiasis in the world due to its vast geographical area, large population and unique eating habits. The tradition of Chinese herbal medicine in the treatment of urolithiasis has a long history in China (13). Relinqing is one of the most widely used drugs for the treatment of urolithiasis in China (14). Polygonum capitatum from Yunnan, a traditional Chinese herbal medicine area in China, has the effects of clearing away heat and detoxifying, diuresis and relieving stranguria, and is used for heat stranguria caused by damp-heat in lower jiao. However, according to our experience, the effect of Relinqing on removing residual stones after endoscopic lithotripsy is limited. TFDS is a newly listed traditional Chinese medicine. It has been proved in previous animal experiments that TFDS can inhibit the formation of renal calcium oxalate stones in rats by reducing the peroxidation damage of renal tubular epithelium, reducing urinary oxalic acid and increasing urinary calcium excretion. It has shown good safety and effectiveness in RCT studies (5, 15). Therefore, we further explored the effect of TFDS in the treatment of residual stones after ureteral calculi in the real world. To be noted, the only indication for TFDS approved by CFDA is to promote the removal of ureteral stones, while the post-operative usage of TFDS still remained to be determined. In this study, it is showed that TFDS played an important role in the process of assisted stone removal after endoscopic treatment of ureteral calculi. The effective rate reached 48% in patients with a median follow-up of 20 days, which was significantly higher than 18% in the control cohort, especially in calcium oxalate stones. However, the deficiency of this study is that the effects of the two drugs were not observed in apatite stones due to the sparse sample size. In addition, because uric acid stones are not visualized on x-rays, we also excluded patients with uric acid stones. In addition, only 48% of the effective rate of TFDS suggested that it could be combined with other drugs for treatment.

With the development of various imaging techniques, the detection effect of residual stones after surgery is getting better and better (16). The stone-free rate is an important indicator for judging the efficacy of surgery. However, researchers are controversial in the definition of stone-free status and imaging evaluation methods. Critical points such as 4 mm, 2 mm or completely invisible stones and imaging methods such as urinary system ultrasonography (USG) and/or kidney-ureter-bladder radiography (KUB), or non-contrast computed tomography (NCCT) have been reported (17, 18). Although KUB can overestimate the postoperative stone clearance rate compared with CT, KUB has the advantages of short examination time, low radiation dose and high economic value (19, 20). In our study, KUB was used in the residual stone cohort the next day after surgery and in the postoperative follow-up, and the results were consistent, so the results were credible. Considering the clinical benefits of patients, all patients with residual stones after surgery were treated with oral drugs without placebo control, which is a flaw in the experimental design and needs further support from prospective studies in the future.

To be noted, there were several limitations should be taken into consideration. Firstly, this study was designed as a retrospective cohort study, and a large-scale prospective randomized control study should be performed to further confirm our findings. Then, the follow-up time should be extended to at least 6 to 12 months to examine the long-term safety of TFDS in this study. Then, residual stones after FURL should be distinctly studied except for TFDS administration.

In conclusion, this study firstly reported a novel treatment protocol for urolithiasis with the combination of dusting FURL and post-operative administration of TFDS. It has been observed that this treatment protocol can effectively treat the urolithiasis with high stone-clearance, and showed highly economic benefits.

## Data Availability

The raw data supporting the conclusions of this article will be made available by the authors, without undue reservation.
