# Supplementary material for: Efficacy and cost-effectiveness analysis of flexible ureteroscopic lithotripsy with TFDS in the treatment of urolithiasis
Source: Front Surg. 2024 Nov 27;11:1489397. doi: 10.3389/fsurg.2024.1489397 (PMC11631858; doi:10.3389/fsurg.2024.1489397)
Supplement: Supplementary file 1 [file Table1.docx]

| Supplemental Table 1. Comparison of demographic characteristics between the two groups in the subgroup analysis with residual stones | | | | |
| --- | --- | --- | --- | --- |
|  | Residual-Control group (n = 22) | Residual-TFDS group (n = 21) | Statistic | *P* |
| Duration to reach the end point,days,M (Q₁, Q₃) | 22.50 (20.00, 31.25) | 20.00 (17.00, 29.00) | Z=-1.33 | 0.184 |
| Follow-up duration, days,M (Q₁, Q₃) | 20.00 (17.00, 29.00) | 22.50 (20.00, 31.25) | Z=-1.33 | 0.184 |
| Age, years old,Mean(SD) | 52.50 (40.25, 60.50) | 54.00 (44.00, 60.00) | Z=-0.24 | 0.808 |
| hospitalization duration, date,M (Q₁, Q₃) | 4.00 (3.25, 6.00) | 4.00 (3.00, 4.00) | Z=-1.36 | 0.172 |
| Total cost during hospitalization, RMB,M (Q₁, Q₃) | 26684.75 (23013.47, 29903.90) | 23957.50 (22587.80, 25114.90) | Z=-1.96 | 0.050 |
| Drug costs, M (Q₁, Q₃) | 519.75 (462.00, 721.88) | 856.80 (728.28, 1242.36) | Z=-3.61 | **<0.001** |
| BMI, M (Q₁, Q₃) | 24.45 (22.05, 26.27) | 24.72 (22.49, 25.71) | Z=-0.23 | 0.817 |
| Operation duration, minute,M (Q₁, Q₃) | 43.00 (28.50, 49.00) | 45.00 (30.00, 56.00) | Z=-0.80 | 0.422 |
| Stone long diameter, mm,M (Q₁, Q₃) | 12.60 (8.70, 16.62) | 8.50 (7.70, 14.10) | Z=-1.49 | 0.135 |
| Stone short diameter, M (Q₁, Q₃) | 8.05 (6.70, 11.07) | 7.00 (6.30, 8.70) | Z=-1.47 | 0.141 |
| Stone longitudinal diameter, M (Q₁, Q₃) | 11.90 (9.43, 15.20) | 13.10 (8.70, 15.80) | Z=-0.12 | 0.903 |
| CT value, Hu,M (Q₁, Q₃) | 1145.00 (1024.25, 1324.00) | 1156.00 (967.00, 1293.00) | Z=-0.32 | 0.752 |
| urinary leukocyte,/μl, M (Q₁, Q₃) | 53.50 (24.75, 133.75) | 80.00 (22.00, 257.00) | Z=-0.23 | 0.817 |
| ALT, M (Q₁, Q₃) | 18.65 (14.32, 28.27) | 20.90 (15.80, 26.20) | Z=-0.94 | 0.350 |
| AST, M (Q₁, Q₃) | 20.10 (18.40, 24.90) | 21.10 (18.30, 27.80) | Z=-0.38 | 0.706 |
| Urea, M (Q₁, Q₃) | 5.81 (4.75, 6.87) | 5.93 (4.62, 6.88) | Z=0.00 | >0.999 |
| Creatinine, M (Q₁, Q₃) | 83.90 (65.75, 88.87) | 73.60 (62.80, 88.50) | Z=-0.74 | 0.459 |
| peripheral blood white blood cells, M (Q₁, Q₃) | 6.24 (5.52, 6.79) | 6.36 (5.71, 7.32) | Z=-0.61 | 0.539 |
| peripheral blood lymphocyte, M (Q₁, Q₃) | 1.99 (1.62, 2.15) | 2.01 (1.69, 2.55) | Z=-1.24 | 0.215 |
| peripheral blood neutrophils, M (Q₁, Q₃) | 3.55 (2.85, 4.21) | 3.52 (3.26, 4.60) | Z=-0.43 | 0.671 |
| Peripheral blood hemoglobin, Mean(SD) | 138.00 (129.50, 148.50) | 143.00 (126.00, 150.00) | Z=-0.51 | 0.610 |
| peripheral platelet, M (Q₁, Q₃) | 221.50 (205.00, 259.00) | 213.00 (193.00, 256.00) | Z=-0.01 | 0.990 |
| Stones disappeared in KUB at follow-up time, n(%) |  |  | χ²=4.24 | **0.039** |
| No | 18 (81.82) | 11 (52.38) |  |  |
| Yes | 4 (18.18) | 10 (47.62) |  |  |
| Sex, n(%) |  |  | χ²=0.07 | 0.795 |
| Female | 6 (27.27) | 5 (23.81) |  |  |
| Male | 16 (72.73) | 16 (76.19) |  |  |
| stone composition, n(%) |  |  | χ²=0.45 | 0.502 |
| Calcium oxalate stone | 16 (72.73) | 18 (85.71) |  |  |
| Apatite stone | 6 (27.27) | 3 (14.29) |  |  |
| Duration of stone disease history,days, n(%) |  |  | - | 0.746 |
| ≤14 | 6 (27.27) | 7 (33.33) |  |  |
| 15-30 | 2 (9.09) | 3 (14.29) |  |  |
| ≥31 | 14 (63.64) | 11 (52.38) |  |  |
| Smoking history, n(%) |  |  | χ²=0.20 | 0.656 |
| No | 18 (81.82) | 15 (71.43) |  |  |
| Yes | 4 (18.18) | 6 (28.57) |  |  |
| Drinking history, n(%) |  |  | χ²=0.45 | 0.502 |
| No | 16 (72.73) | 18 (85.71) |  |  |
| Yes | 6 (27.27) | 3 (14.29) |  |  |
| history of hypertension, n(%) |  |  | χ²=1.36 | 0.243 |
| No | 19 (86.36) | 14 (66.67) |  |  |
| Yes | 3 (13.64) | 7 (33.33) |  |  |
| history of diabetes, n(%) |  |  | - | >0.999 |
| No | 21 (95.45) | 21 (100.00) |  |  |
| Yes | 1 (4.55) | 0 (0.00) |  |  |
| History of cancer, n(%) |  |  | χ²=0.23 | 0.634 |
| No | 19 (86.36) | 20 (95.24) |  |  |
| Yes | 3 (13.64) | 1 (4.76) |  |  |
| History of ESWL, n(%) |  |  | χ²=0.00 | >0.999 |
| No | 18 (81.82) | 18 (85.71) |  |  |
| Yes | 4 (18.18) | 3 (14.29) |  |  |
| History of urinary surgery, n(%) |  |  | χ²=0.19 | 0.665 |
| No | 16 (72.73) | 14 (66.67) |  |  |
| Yes | 6 (27.27) | 7 (33.33) |  |  |
| Surgical history of ureteroscopy on the operative side, n(%) |  |  | χ²=0.14 | 0.705 |
| No | 18 (81.82) | 19 (90.48) |  |  |
| Yes | 4 (18.18) | 2 (9.52) |  |  |
| Surgical history of ureteral dilatation on the operative side, n(%) |  |  | - | >0.999 |
| No | 21 (95.45) | 20 (95.24) |  |  |
| Yes | 1 (4.55) | 1 (4.76) |  |  |
| Surgical history of ureterolithotomy on the surgical side, n(%) |  |  | - | 0.488 |
| No | 22 (100.00) | 20 (95.24) |  |  |
| Yes | 0 (0.00) | 1 (4.76) |  |  |
| History of percutaneous nephrolithotomy on the surgical side, n(%) |  |  | χ²=0.00 | >0.999 |
| No | 20 (90.91) | 20 (95.24) |  |  |
| Yes | 2 (9.09) | 1 (4.76) |  |  |
| Ureteral stenosis on the operative side, n(%) |  |  | χ²=0.00 | >0.999 |
| No | 19 (86.36) | 18 (85.71) |  |  |
| Yes | 3 (13.64) | 3 (14.29) |  |  |
| Surgical side, n(%) |  |  | χ²=0.72 | 0.396 |
| Left | 13 (59.09) | 15 (71.43) |  |  |
| Right | 9 (40.91) | 6 (28.57) |  |  |
| Placed Ureteral stent before operation., n(%) |  |  | χ²=0.00 | >0.999 |
| No | 19 (86.36) | 18 (85.71) |  |  |
| Yes | 3 (13.64) | 3 (14.29) |  |  |
| The location of stones on CT, n(%) |  |  | - | 0.532 |
| Kidney | 11 (50.00) | 9 (42.86) |  |  |
| Ureter | 3 (13.64) | 6 (28.57) |  |  |
| Kidney and ureter | 8 (36.36) | 6 (28.57) |  |  |
| Results of preoperative bacterial culture, n(%) |  |  | χ²=0.00 | >0.999 |
| Without bacteria | 20 (90.91) | 19 (90.48) |  |  |
| with bacteria | 2 (9.09) | 2 (9.52) |  |  |
| Leukocyte esterase in urine, n(%) |  |  | - | 0.962 |
| negative | 13 (59.09) | 11 (52.38) |  |  |
| 1+ | 4 (18.18) | 4 (19.05) |  |  |
| 2+ | 1 (4.55) | 2 (9.52) |  |  |
| 3+ | 4 (18.18) | 4 (19.05) |  |  |
| Urine bacterial smear, n(%) |  |  | - | 0.867 |
| negative | 18 (81.82) | 16 (76.19) |  |  |
| 1+ | 3 (13.64) | 2 (9.52) |  |  |
| 2+ | 0 (0.00) | 1 (4.76) |  |  |
| 3+ | 0 (0.00) | 1 (4.76) |  |  |
| 4+ | 1 (4.55) | 1 (4.76) |  |  |
