# Supplementary material for: Efficacy and cost-effectiveness analysis of flexible ureteroscopic lithotripsy with TFDS in the treatment of urolithiasis
Source: Front Surg. 2024 Nov 27;11:1489397. doi: 10.3389/fsurg.2024.1489397 (PMC11631858; doi:10.3389/fsurg.2024.1489397)
Supplement: Supplementary file 2 [file Table2.docx]

| Supplemental Table 2. Comparison of demographic characteristics between the two groups in the in the calcium oxalate stone subgroup analysis with residual stones | | | | | |
| --- | --- | --- | --- | --- | --- |
|  | Total (n = 34) | Control group (n = 16) | TFDS group (n = 18) | Statistic | *P* |
| Duration to reach the end point,days,M (Q₁, Q₃) | 22.00 (17.00, 30.50) | 27.50 (19.25, 32.00) | 20.00 (17.00, 29.00) | Z=-1.13 | 0.26 |
| Follow-up duration, days,Mean ± SD | 24.24 ± 9.11 | 26.00 ± 9.14 | 22.67 ± 9.06 | t=1.07 | 0.294 |
| Age, years old,M (Q₁, Q₃) | 56.00 (44.00, 61.00) | 57.00 (43.25, 62.75) | 55.00 (45.00, 60.75) | Z=-0.33 | 0.743 |
| hospitalization duration, date,M (Q₁, Q₃) | 4.00 (3.00, 5.00) | 4.00 (3.75, 6.00) | 4.00 (3.00, 4.00) | Z=-1.51 | 0.131 |
| Total cost during hospitalization, RMB,M (Q₁, Q₃) | 24740.60 (22861.33, 27575.22) | 27195.30 (23515.43, 30544.67) | 24055.15 (21640.70, 25413.55) | Z=-1.64 | 0.102 |
| BMI, M (Q₁, Q₃) | 24.60 (21.90, 26.35) | 24.01 (21.88, 26.56) | 24.60 (22.03, 25.59) | Z=0.00 | 1 |
| Operation duration, minute,M (Q₁, Q₃) | 43.00 (30.00, 53.75) | 43.00 (30.00, 47.00) | 42.50 (29.25, 58.00) | Z=-0.28 | 0.782 |
| Stone long diameter, mm,M (Q₁, Q₃) | 10.45 (8.25, 15.45) | 13.40 (8.42, 16.65) | 9.50 (8.25, 15.00) | Z=-1.00 | 0.317 |
| Stone short diameter, M (Q₁, Q₃) | 7.55 (6.43, 10.15) | 7.90 (6.57, 11.03) | 7.10 (6.25, 8.78) | Z=-0.71 | 0.479 |
| Stone longitudinal diameter, M (Q₁, Q₃) | 12.80 (8.85, 15.73) | 11.45 (8.98, 14.60) | 13.35 (9.20, 17.15) | Z=-0.62 | 0.534 |
| CT value, Hu,M (Q₁, Q₃) | 1167.50 (1085.25, 1324.00) | 1227.50 (1088.50, 1336.25) | 1151.50 (978.00, 1225.00) | Z=-0.97 | 0.33 |
| urinary leukocyte,/μl, M (Q₁, Q₃) | 59.50 (22.25, 133.75) | 52.00 (22.00, 108.25) | 67.50 (23.25, 240.25) | Z=-0.50 | 0.617 |
| ALT, M (Q₁, Q₃) | 19.55 (15.03, 25.05) | 18.90 (14.78, 26.02) | 20.40 (15.80, 24.75) | Z=-0.29 | 0.769 |
| AST, M (Q₁, Q₃) | 20.80 (18.40, 24.90) | 21.30 (18.78, 25.48) | 20.30 (17.85, 23.32) | Z=-0.47 | 0.641 |
| Urea, M (Q₁, Q₃) | 6.01 (4.62, 7.12) | 5.98 (4.98, 7.00) | 6.27 (4.55, 7.18) | Z=-0.02 | 0.986 |
| Creatinine, M (Q₁, Q₃) | 82.00 (66.27, 88.95) | 83.90 (74.60, 88.43) | 76.10 (60.82, 103.57) | Z=-0.47 | 0.641 |
| peripheral blood white blood cells, M (Q₁, Q₃) | 6.10 (5.48, 7.24) | 5.79 (5.49, 6.78) | 6.64 (5.46, 7.47) | Z=-0.97 | 0.33 |
| peripheral blood lymphocyte, M (Q₁, Q₃) | 2.01 (1.62, 2.29) | 2.01 (1.61, 2.12) | 2.00 (1.68, 2.49) | Z=-0.81 | 0.417 |
| peripheral blood neutrophils, M (Q₁, Q₃) | 3.39 (2.85, 4.46) | 3.27 (2.82, 3.88) | 3.66 (3.28, 4.73) | Z=-1.22 | 0.221 |
| Peripheral blood hemoglobin, Mean(SD) | 141.00 (129.50, 148.75) | 141.00 (130.50, 147.50) | 141.00 (127.25, 148.75) | Z=-0.16 | 0.876 |
| peripheral platelet, M (Q₁, Q₃) | 218.50 (200.50, 259.00) | 221.50 (205.00, 257.00) | 215.00 (194.50, 264.25) | Z=-0.05 | 0.959 |
| Stones disappeared in KUB at follow-up time, n(%) |  |  |  | - | **0.003** |
| No | 23 (67.65) | 15 (93.75) | 8 (44.44) |  |  |
| Yes | 11 (32.35) | 1 (6.25) | 10 (55.56) |  |  |
| Sex, n(%) |  |  |  | - | 1 |
| Female | 7 (20.59) | 3 (18.75) | 4 (22.22) |  |  |
| Male | 27 (79.41) | 13 (81.25) | 14 (77.78) |  |  |
| Duration of stone disease history,days, n(%) |  |  |  | - | 0.885 |
| ≤14 | 10 (29.41) | 4 (25.00) | 6 (33.33) |  |  |
| 15-30 | 4 (11.76) | 2 (12.50) | 2 (11.11) |  |  |
| ≥31 | 20 (58.82) | 10 (62.50) | 10 (55.56) |  |  |
| Smoking history, n(%) |  |  |  | - | 0.693 |
| No | 26 (76.47) | 13 (81.25) | 13 (72.22) |  |  |
| Yes | 8 (23.53) | 3 (18.75) | 5 (27.78) |  |  |
| Drinking history, n(%) |  |  |  | - | 0.11 |
| No | 26 (76.47) | 10 (62.50) | 16 (88.89) |  |  |
| Yes | 8 (23.53) | 6 (37.50) | 2 (11.11) |  |  |
| history of hypertension, n(%) |  |  |  | - | 0.233 |
| No | 26 (76.47) | 14 (87.50) | 12 (66.67) |  |  |
| Yes | 8 (23.53) | 2 (12.50) | 6 (33.33) |  |  |
| history of diabetes, n(%) |  |  |  | - | 0.471 |
| No | 33 (97.06) | 15 (93.75) | 18 (100.00) |  |  |
| Yes | 1 (2.94) | 1 (6.25) | 0 (0.00) |  |  |
| History of cancer, n(%) |  |  |  | - | 0.323 |
| No | 30 (88.24) | 13 (81.25) | 17 (94.44) |  |  |
| Yes | 4 (11.76) | 3 (18.75) | 1 (5.56) |  |  |
| History of ESWL, n(%) |  |  |  | - | 0.681 |
| No | 27 (79.41) | 12 (75.00) | 15 (83.33) |  |  |
| Yes | 7 (20.59) | 4 (25.00) | 3 (16.67) |  |  |
| History of urinary surgery, n(%) |  |  |  | - | 0.693 |
| No | 26 (76.47) | 13 (81.25) | 13 (72.22) |  |  |
| Yes | 8 (23.53) | 3 (18.75) | 5 (27.78) |  |  |
| Surgical history of ureteroscopy on the operative side, n(%) |  |  |  | - | 1 |
| No | 30 (88.24) | 14 (87.50) | 16 (88.89) |  |  |
| Yes | 4 (11.76) | 2 (12.50) | 2 (11.11) |  |  |
| Surgical history of ureteral dilatation on the operative side, n(%) |  |  |  | - | 1 |
| No | 32 (94.12) | 15 (93.75) | 17 (94.44) |  |  |
| Yes | 2 (5.88) | 1 (6.25) | 1 (5.56) |  |  |
| Surgical history of ureterolithotomy on the surgical side, n(%) |  |  |  | - | 1 |
| No | 33 (97.06) | 16 (100.00) | 17 (94.44) |  |  |
| Yes | 1 (2.94) | 0 (0.00) | 1 (5.56) |  |  |
| History of percutaneous nephrolithotomy on the surgical side, n(%) |  |  |  | - | 1 |
| No | 33 (97.06) | 16 (100.00) | 17 (94.44) |  |  |
| Yes | 1 (2.94) | 0 (0.00) | 1 (5.56) |  |  |
| Ureteral stenosis on the operative side, n(%) |  |  |  | - | 1 |
| No | 29 (85.29) | 14 (87.50) | 15 (83.33) |  |  |
| Yes | 5 (14.71) | 2 (12.50) | 3 (16.67) |  |  |
| Surgical side, n(%) |  |  |  | - | 0.475 |
| Left | 22 (64.71) | 9 (56.25) | 13 (72.22) |  |  |
| Right | 12 (35.29) | 7 (43.75) | 5 (27.78) |  |  |
| Placed Ureteral stent before operation., n(%) |  |  |  | - | 0.323 |
| No | 30 (88.24) | 13 (81.25) | 17 (94.44) |  |  |
| Yes | 4 (11.76) | 3 (18.75) | 1 (5.56) |  |  |
| The location of stones on CT, n(%) |  |  |  | - | 0.552 |
| Kidney | 16 (47.06) | 8 (50.00) | 8 (44.44) |  |  |
| Ureter | 7 (20.59) | 2 (12.50) | 5 (27.78) |  |  |
| Kidney and ureter | 11 (32.35) | 6 (37.50) | 5 (27.78) |  |  |
| Results of preoperative bacterial culture, n(%) |  |  |  | - | 1 |
| Without bacteria | 31 (91.18) | 15 (93.75) | 16 (88.89) |  |  |
| with bacteria | 3 (8.82) | 1 (6.25) | 2 (11.11) |  |  |
| Leukocyte esterase in urine, n(%) |  |  |  | - | 0.88 |
| negative | 20 (58.82) | 10 (62.50) | 10 (55.56) |  |  |
| 1+ | 8 (23.53) | 4 (25.00) | 4 (22.22) |  |  |
| 2+ | 2 (5.88) | 1 (6.25) | 1 (5.56) |  |  |
| 3+ | 4 (11.76) | 1 (6.25) | 3 (16.67) |  |  |
| Urine bacterial smear, n(%) |  |  |  | - | 0.891 |
| negative | 28 (82.35) | 13 (81.25) | 15 (83.33) |  |  |
| 1+ | 3 (8.82) | 2 (12.50) | 1 (5.56) |  |  |
| 2+ | 0(0.00) | 0(0.00) | 0(0.00) |  |  |
| 3+ | 1 (2.94) | 0 (0.00) | 1 (5.56) |  |  |
| 4+ | 2 (5.88) | 1 (6.25) | 1 (5.56) |  |  |
