# Supplementary material for: Efficacy and cost-effectiveness analysis of flexible ureteroscopic lithotripsy with TFDS in the treatment of urolithiasis
Source: Front Surg. 2024 Nov 27;11:1489397. doi: 10.3389/fsurg.2024.1489397 (PMC11631858; doi:10.3389/fsurg.2024.1489397)
Supplement: Supplementary file 3 [file Table3.docx]

| Supplemental Table 3. Intergroup comparison of different outcomes in the calcium oxalate residual stone cohort | | | | | |
| --- | --- | --- | --- | --- | --- |
| Variable | Total (n = 34) | residual stone group (n = 23) | non-residual stone group (n = 11) | Statistic | *P* |
| Duration to reach the end point,days,M (Q₁, Q₃) | 22.00 (17.00, 30.50) | 26.00 (20.00, 31.00) | 18.00 (17.00, 25.50) | Z=-1.16 | 0.245 |
| Follow-up duration, days,M (Q₁, Q₃) | 22.00 (17.00, 30.50) | 26.00 (20.00, 31.00) | 18.00 (17.00, 25.50) | Z=-1.16 | 0.245 |
| Age, years old,M (Q₁, Q₃) | 56.00 (44.00, 61.00) | 56.00 (48.00, 61.00) | 58.00 (41.00, 64.00) | Z=-0.06 | 0.956 |
| hospitalization duration, date,M (Q₁, Q₃) | 4.00 (3.00, 5.00) | 4.00 (3.00, 5.00) | 4.00 (3.00, 4.00) | Z=-0.52 | 0.605 |
| BMI, M (Q₁, Q₃) | 24.60 (21.90, 26.35) | 22.88 (21.74, 25.95) | 25.25 (24.73, 26.14) | Z=-1.51 | 0.131 |
| Operation duration, minute,M (Q₁, Q₃) | 43.00 (30.00, 53.75) | 45.00 (31.50, 58.50) | 30.00 (26.00, 47.00) | Z=-1.59 | 0.113 |
| Stone long diameter, mm,M (Q₁, Q₃) | 10.45 (8.25, 15.45) | 14.90 (8.45, 18.85) | 8.50 (7.70, 10.35) | Z=-1.93 | 0.053 |
| Stone short diameter, M (Q₁, Q₃) | 7.55 (6.43, 10.15) | 7.90 (6.65, 11.05) | 7.20 (5.30, 8.35) | Z=-1.40 | 0.162 |
| Stone longitudinal diameter, M (Q₁, Q₃) | 12.80 (8.85, 15.73) | 12.50 (9.00, 18.25) | 13.20 (9.30, 14.05) | Z=-0.74 | 0.462 |
| CT value, Hu,M (Q₁, Q₃) | 1167.50 (1085.25, 1324.00) | 1217.00 (1088.00, 1381.00) | 1132.00 (989.00, 1194.50) | Z=-1.54 | 0.123 |
| urinary leukocyte,/μl, M (Q₁, Q₃) | 59.50 (22.25, 133.75) | 64.00 (25.00, 100.00) | 55.00 (16.00, 754.50) | Z=-0.31 | 0.754 |
| ALT, M (Q₁, Q₃) | 19.55 (15.03, 25.05) | 22.20 (15.05, 27.80) | 17.90 (15.10, 21.75) | Z=-1.05 | 0.294 |
| AST, M (Q₁, Q₃) | 20.80 (18.40, 24.90) | 22.10 (18.95, 28.65) | 18.80 (17.40, 21.60) | Z=-2.08 | **0.037** |
| Urea, M (Q₁, Q₃) | 6.01 (4.62, 7.12) | 6.50 (5.21, 7.06) | 4.65 (4.58, 7.08) | Z=-0.54 | 0.586 |
| Creatinine, M (Q₁, Q₃) | 82.00 (66.27, 88.95) | 84.60 (67.45, 92.45) | 73.60 (64.20, 85.35) | Z=-0.88 | 0.377 |
| peripheral blood white blood cells, M (Q₁, Q₃) | 6.10 (5.48, 7.24) | 6.08 (5.60, 6.81) | 6.90 (5.25, 7.80) | Z=-0.00 | 1 |
| peripheral blood lymphocyte, M (Q₁, Q₃) | 2.01 (1.62, 2.29) | 2.01 (1.62, 2.23) | 2.01 (1.69, 2.43) | Z=-0.52 | 0.606 |
| peripheral blood neutrophils, M (Q₁, Q₃) | 3.39 (2.85, 4.46) | 3.35 (2.87, 3.92) | 4.42 (2.98, 4.80) | Z=-0.79 | 0.429 |
| Peripheral blood hemoglobin, Mean(SD) | 141.00 (129.50, 148.75) | 142.00 (131.00, 149.00) | 136.00 (116.00, 148.00) | Z=-1.22 | 0.224 |
| peripheral platelet, M (Q₁, Q₃) | 218.50 (200.50, 259.00) | 225.00 (205.00, 258.00) | 210.00 (182.00, 285.50) | Z=-0.33 | 0.74 |
| drug category,n(%) |  |  |  | - | **0.003** |
| None | 16 (47.06) | 15 (65.22) | 1 (9.09) |  |  |
| Guangshitong | 18 (52.94) | 8 (34.78) | 10 (90.91) |  |  |
| Sex, n(%) |  |  |  | - | 0.178 |
| Female | 7 (20.59) | 3 (13.04) | 4 (36.36) |  |  |
| Male | 27 (79.41) | 20 (86.96) | 7 (63.64) |  |  |
| Duration of stone disease history,days, n(%) |  |  |  | - | 0.059 |
| ≤14 | 10 (29.41) | 4 (17.39) | 6 (54.55) |  |  |
| 15-30 | 4 (11.76) | 4 (17.39) | 0 (0.00) |  |  |
| ≥31 | 20 (58.82) | 15 (65.22) | 5 (45.45) |  |  |
| Smoking history, n(%) |  |  |  | - | 0.227 |
| No | 26 (76.47) | 16 (69.57) | 10 (90.91) |  |  |
| Yes | 8 (23.53) | 7 (30.43) | 1 (9.09) |  |  |
| Drinking history, n(%) |  |  |  | - | 0.227 |
| No | 26 (76.47) | 16 (69.57) | 10 (90.91) |  |  |
| Yes | 8 (23.53) | 7 (30.43) | 1 (9.09) |  |  |
| history of hypertension, n(%) |  |  |  | - | 0.388 |
| No | 26 (76.47) | 19 (82.61) | 7 (63.64) |  |  |
| Yes | 8 (23.53) | 4 (17.39) | 4 (36.36) |  |  |
| history of diabetes, n(%) |  |  |  | - | 1 |
| No | 33 (97.06) | 22 (95.65) | 11 (100.00) |  |  |
| Yes | 1 (2.94) | 1 (4.35) | 0 (0.00) |  |  |
| History of cancer, n(%) |  |  |  | - | 0.58 |
| No | 30 (88.24) | 21 (91.30) | 9 (81.82) |  |  |
| Yes | 4 (11.76) | 2 (8.70) | 2 (18.18) |  |  |
| History of ESWL, n(%) |  |  |  | - | 1 |
| No | 27 (79.41) | 18 (78.26) | 9 (81.82) |  |  |
| Yes | 7 (20.59) | 5 (21.74) | 2 (18.18) |  |  |
| History of urinary surgery, n(%) |  |  |  | - | 0.227 |
| No | 26 (76.47) | 16 (69.57) | 10 (90.91) |  |  |
| Yes | 8 (23.53) | 7 (30.43) | 1 (9.09) |  |  |
| Surgical history of ureteroscopy on the operative side, n(%) |  |  |  | - | 0.28 |
| No | 30 (88.24) | 19 (82.61) | 11 (100.00) |  |  |
| Yes | 4 (11.76) | 4 (17.39) | 0 (0.00) |  |  |
| Surgical history of ureteral dilatation on the operative side, n(%) |  |  |  | - | 1 |
| No | 32 (94.12) | 21 (91.30) | 11 (100.00) |  |  |
| Yes | 2 (5.88) | 2 (8.70) | 0 (0.00) |  |  |
| Surgical history of ureterolithotomy on the surgical side, n(%) |  |  |  | - | 1 |
| No | 33 (97.06) | 22 (95.65) | 11 (100.00) |  |  |
| Yes | 1 (2.94) | 1 (4.35) | 0 (0.00) |  |  |
| History of percutaneous nephrolithotomy on the surgical side, n(%) |  |  |  | - | 1 |
| No | 33 (97.06) | 22 (95.65) | 11 (100.00) |  |  |
| Yes | 1 (2.94) | 1 (4.35) | 0 (0.00) |  |  |
| Ureteral stenosis on the operative side, n(%) |  |  |  | - | 1 |
| No | 29 (85.29) | 19 (82.61) | 10 (90.91) |  |  |
| Yes | 5 (14.71) | 4 (17.39) | 1 (9.09) |  |  |
| Surgical side, n(%) |  |  |  | - | 0.705 |
| Left | 22 (64.71) | 14 (60.87) | 8 (72.73) |  |  |
| Right | 12 (35.29) | 9 (39.13) | 3 (27.27) |  |  |
| Placed Ureteral stent before operation., n(%) |  |  |  | - | 0.28 |
| No | 30 (88.24) | 19 (82.61) | 11 (100.00) |  |  |
| Yes | 4 (11.76) | 4 (17.39) | 0 (0.00) |  |  |
| The location of stones on CT, n(%) |  |  |  | - | 0.709 |
| Kidney | 16 (47.06) | 12 (52.17) | 4 (36.36) |  |  |
| Ureter | 7 (20.59) | 4 (17.39) | 3 (27.27) |  |  |
| Kidney and ureter | 11 (32.35) | 7 (30.43) | 4 (36.36) |  |  |
| Results of preoperative bacterial culture, n(%) |  |  |  | - | 0.239 |
| Without bacteria | 31 (91.18) | 22 (95.65) | 9 (81.82) |  |  |
| with bacteria | 3 (8.82) | 1 (4.35) | 2 (18.18) |  |  |
| Leukocyte esterase in urine, n(%) |  |  |  | - | 0.276 |
| negative | 20 (58.82) | 14 (60.87) | 6 (54.55) |  |  |
| 1+ | 8 (23.53) | 6 (26.09) | 2 (18.18) |  |  |
| 2+ | 2 (5.88) | 2 (8.70) | 0 (0.00) |  |  |
| 3+ | 4 (11.76) | 1 (4.35) | 3 (27.27) |  |  |
| Urine bacterial smear, n(%) |  |  |  | - | **0.042** |
| negative | 28 (82.35) | 20 (86.96) | 8 (72.73) |  |  |
| 1+ | 3 (8.82) | 3 (13.04) | 0 (0.00) |  |  |
| 2+ | 0(0.00) | 0(0.00) | 0(0.00) |  |  |
| 3+ | 1 (2.94) | 0 (0.00) | 1 (9.09) |  |  |
| 4+ | 2 (5.88) | 0 (0.00) | 2 (18.18) |  |  |
