# Supplementary material for: Efficacy and cost-effectiveness analysis of flexible ureteroscopic lithotripsy with TFDS in the treatment of urolithiasis
Source: Front Surg. 2024 Nov 27;11:1489397. doi: 10.3389/fsurg.2024.1489397 (PMC11631858; doi:10.3389/fsurg.2024.1489397)
Supplement: Supplementary file 4 [file Table4.docx]

| Supplemental Table 4. Multivariate logistic regression analysis of factors affecting stone clearance rate in the calcium oxalate residual stone cohort | | | | | |
| --- | --- | --- | --- | --- | --- |
| Variables | β | S.E | Z | *P* | OR (95%CI) |
| Intercept | 0.99 | 2.47 | 0.4 | 0.688 | 2.70 (0.02 ~ 342.75) |
| AST, M (Q₁, Q₃) | -0.18 | 0.11 | -1.54 | 0.123 | 0.84 (0.67 ~ 1.05) |
| drug category,n(%) |  |  |  |  |  |
| Relinqing |  |  |  |  | 1.00 (Reference) |
| TFDS | 3.14 | 1.19 | 2.64 | **0.008** | 23.12 (2.24 ~ 238.89) |
