# Supplementary material for: Efficacy and cost-effectiveness analysis of flexible ureteroscopic lithotripsy with TFDS in the treatment of urolithiasis
Source: Front Surg. 2024 Nov 27;11:1489397. doi: 10.3389/fsurg.2024.1489397 (PMC11631858; doi:10.3389/fsurg.2024.1489397)
Supplement: Supplementary file 5 [file Table5.docx]

| Supplemental Table 5. Sensitivity analysis of cost effective analysis | | | | | | | |
| --- | --- | --- | --- | --- | --- | --- | --- |
|  | number of samples | Median cost discount 80 %（C）/RMB | Effectiveness（E）/% | C/E | △C | △E | △C/△E |
| Control group | 22 | 415.8 | 18.18 | 22.87 |  |  |  |
| TGDS group | 21 | 685.44 | 47.62 | 14.39 | 269.64 | 29.44 | 9.16 |
